# ClpB mutants of *Francisella tularensis* subspecies *holarctica* and *tularensis* are defective for type VI secretion and intracellular replication

**DOI:** 10.1038/s41598-018-29745-4

**Published:** 2018-07-27

**Authors:** Athar Alam, Igor Golovliov, Eram Javed, Anders Sjöstedt

**Affiliations:** Department of Clinical Microbiology, Umeå University, SE-901 85 Umeå, Sweden

## Abstract

*Francisella tularensis*, a highly infectious, intracellular bacterium possesses an atypical type VI secretion system (T6SS), which is essential for the virulence of the bacterium. Recent data suggest that the HSP100 family member, ClpB, is involved in T6SS disassembly in the subspecies *Francisella novicida*. Here, we investigated the role of ClpB for the function of the T6SS and for phenotypic characteristics of the human pathogenic subspecies *holarctica* and *tularensis*. The *∆clpB* mutants of the human live vaccine strain, LVS, belonging to subspecies *holarctica*, and the highly virulent SCHU S4 strain, belonging to subspecies *tularensis*, both showed extreme susceptibility to heat shock and low pH, severely impaired type VI secretion (T6S), and significant, but impaired intracellular replication compared to the wild-type strains. Moreover, they showed essentially intact phagosomal escape. Infection of mice demonstrated that both Δ*clpB* mutants were highly attenuated, but the SCHU S4 mutant showed more effective replication than the LVS strain. Collectively, our data demonstrate that ClpB performs multiple functions in the *F*. *tularensis* subspecies *holarctica* and *tularensis* and its function is important for T6S, intracellular replication, and virulence.

## Introduction

The zoonotic disease tularemia is caused by the virulent, intracellular, Gram-negative coccobacillus designated *Francisella tularensis*^1^. Infection can occur through a variety of entry sites, including ingestion, direct contact with non-intact skin or mucous membranes, inhalation, or via the bite of a tick or fly vector^2^. Strains virulent for humans belong to the subspecies *holarctica* and *tularensis*, both of which are highly contagious and the latter subspecies may cause fatal human infection, whereas subspecies *holarctica* cause potentially serious, but not fatal disease. The pathogenicity of *Francisella* is intimately dependent on the *Francisella* Pathogenicity Island (FPI), a gene cluster encoding a functional, but atypical type VI secretion system (T6SS)^3,4^.

The T6SSs are transenvelope complexes specialized in the delivery of effector proteins directly to the target cell membranes of both bacterial and eukaryotic cells, thereby modulating the host-bacterial or bacterial-bacterial interactions^5,6^. The highly dynamic assembly of T6SS starts by formation of a membrane complex^7^ that recruits a baseplate complex^8–11^. At the assembled baseplate, the polymerization of a long tube is initiated, which then becomes wrapped by a sheath^12–15^. The spike and tube then combine with effector molecules, which are exerted upon sheath contraction^15–18^.

The FPI gene cluster of *Francisella* encodes 17 proteins, most of which are required for phagosomal escape and survival inside the host and 8 of them have low sequence similarity to canonical T6SS proteins^19–22^. Recently, a mesh-like structure was observed in *F*. *novicida* and, despite low sequence similarity, its sheath is similar to the contractile sheath of canonical T6SS, indicating the presence of a functional T6SS^14,23,24^. Techniques have been implemented to detect secretion of FPI proteins and several secreted proteins have been identified, although much remains to be understood about their functions, *e*.*g*., no effector function has been assigned to any FPI protein^25–27^. Despite the presence of many functional T6SS homologues, *Francisella* lacks the two ATPases, IcmF/TssM and ClpV, both of which may provide the energy required for secretion in prototypical T6SS^28,29^. An IcmF homologue (termed PdpB) is present, but lacks the Walker A motif necessary for the ATPase activity^22^. *Campylobacter jejuni*, *Helicobacter hepaticus*, and *Salmonella choleraesuis* also lack the ClpV homologue, but demonstrate a functional T6SS^30–33^, indicating that ClpV is not essential for T6S of all the species. Instead, a related member of the ClpV family, the ClpB ATPase, may substitute. The hexameric ClpB molecular chaperone belongs to the ring-forming Clp/Hsp100 proteins^34^, which form two distinct subfamilies; class I proteins, ClpA, ClpB and ClpV, and class II proteins, ClpX and HslU^29,34^. ClpB confers thermotolerance to a range of species via its unfoldase activity^35^, a role executed jointly with the co-chaperones DnaK, DnaJ, and GrpE^36^. ClpV, although being a class I Clp/Hsp100 protein, is not involved in thermotolerance, however, it has been identified *in vitro*^37^ and *in vivo*^18^ as crucial for the disassembly of the contractile sheath in prototypical T6SS^37^. This disassembly activity contributes to the reassembly of an extended sheath^28,38^. Recently, colocalization of ClpB with the contracted sheath of *Francisella novicida* T6SS has been demonstrated and it has been suggested to play an essential role for sheath disassembly^18,23^. Although experimental evidence is lacking, ClpB may also provide energy for the translocation of the T6SS substrate molecules. However, in the absence of ClpB, the assembly is still partially active, demonstrating that its role for T6S is not essential^28,38^.

The Δ*clpB* mutant of the highly virulent strain SCHU S4 of *F*. *tularensis* subspecies *tularensis* has been extensively studied since it is highly attenuated and confers very effective protection in the mouse against challenge with virulent *F*. *tularensis* strains^39–41^. There are several characterized Δ*clpB* mutant of subspecies *holarctica* described. One was derived from a Swedish patient isolate and found to be more attenuated, yet, confer superior protection compared to the human live vaccine strain of the same subspecies^42^. The latter strain was empirically derived from a Russian patient isolate and subsequently passaged in the US and designated the live vaccine strain, LVS^43^. It was tested extensively in human volunteers during the 1960s^44^ and also utilized to protect laboratory staff. It led to an almost 90% reduction of laboratory-acquired tularemia^45^. A Δ*clpB* mutant of the LVS strain has been characterized and observed to induce a more robust proinflammatory response than did the parental strain^46^.

In the present study, we demonstrate that the *∆clpB* mutants of the LVS strain, subspecies *holarctica*, and the highly virulent SCHU S4 strain, subspecies *tularensis*, both are exquisitely susceptible to heat shock and low pH, show defective intracellular growth, concomitantly with impaired T6S.

## Results

### Δ*clpB* mutants are highly susceptible to heat shock and low pH

Survival of Δ*clpB* mutants of any bacterial species is severely compromised during heat stress, since resolubilization of protein aggregates that result from the stress is predominantly dependent on ClpB. To determine the role of ClpB protein of *F*. *tularensis* in stress tolerance, we monitored the survival of LVS and the SCHU S4 Δ*clpB* mutants under various stress conditions.

**Figure 1:**
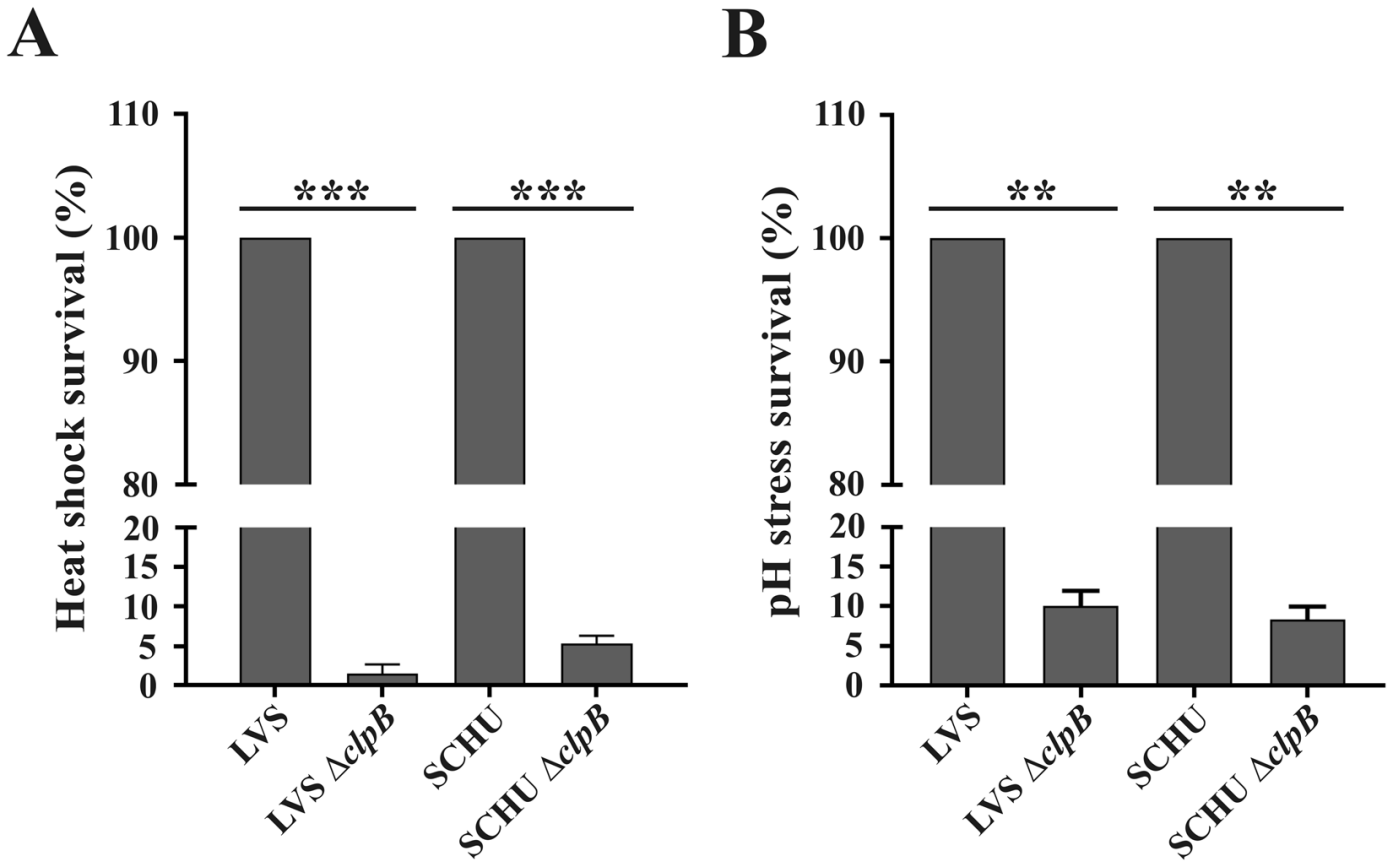
Survival of indicated *F*. *tularensis* strains during heat and pH stress. Overnight grown bacteria were diluted in fresh growth media and subjected to stress at 50 °C for 30 min (**A**) or pH 4.5 for 1 h (**B**). Survival of each strain was monitored as viable bacteria and plotted as percentage of survival, setting wild-type strains survival as 100%. There was no significant killing of any wild-type strain during the treatment. Data are the average survival percentage for at least three independent experiments for each condition. Values are mean ± SD. **P < 0.01; *** indicates *P* < 0.001 *vs*. the corresponding wild-type strain.

When subjected to high temperature (50 °C), as expected, the LVS and SCHU S4 Δ*clpB* mutants showed compromised survival and their numbers decreased to 1.5% and 5.3%, respectively, of the numbers of the wild-type strains after 30 min (Fig. 1A) while the CFU of their wild-type strains, 8.4 and 8.9 log_10_, respectively, did not drop significantly.

We next evaluated the susceptibility of the Δ*clpB* mutant to low pH by exposing bacteria to a pH of 4.5 for 1 h. As in the case of the heat stress response, each of the two wild-type strains was relatively unaffected, while each of the Δ*clpB* mutants was highly susceptible as their survival was less than 10% of the corresponding wild-type strains (*P* < 0.001; Fig. 1B).

The responses of the Δ*clpB* mutants to other forms of stress were also investigated. O_2_^−^ is continuously generated as a by-product of the respiratory chain during bacterial growth. To investigate the resistance to ROS, bacteria were exposed to either paraquat in a disc diffusion assay, or to SIN-1. The former catalyzes the formation of O_2_^−^, whereas the latter is a surrogate for ONOO^−^ susceptibility, which is bactericidal against *F*. *tularensis* in activated macrophages^47^. Both the LVS and SCHU S4 Δ*clpB* mutants and wild-type strains showed the same susceptibility to 40 mM of paraquat or 0.40 mM of SIN-1 (data not shown).

### The *F*. *tularensis* Δ*clpB* mutants show compromised intracellular replication

**Figure 2:**
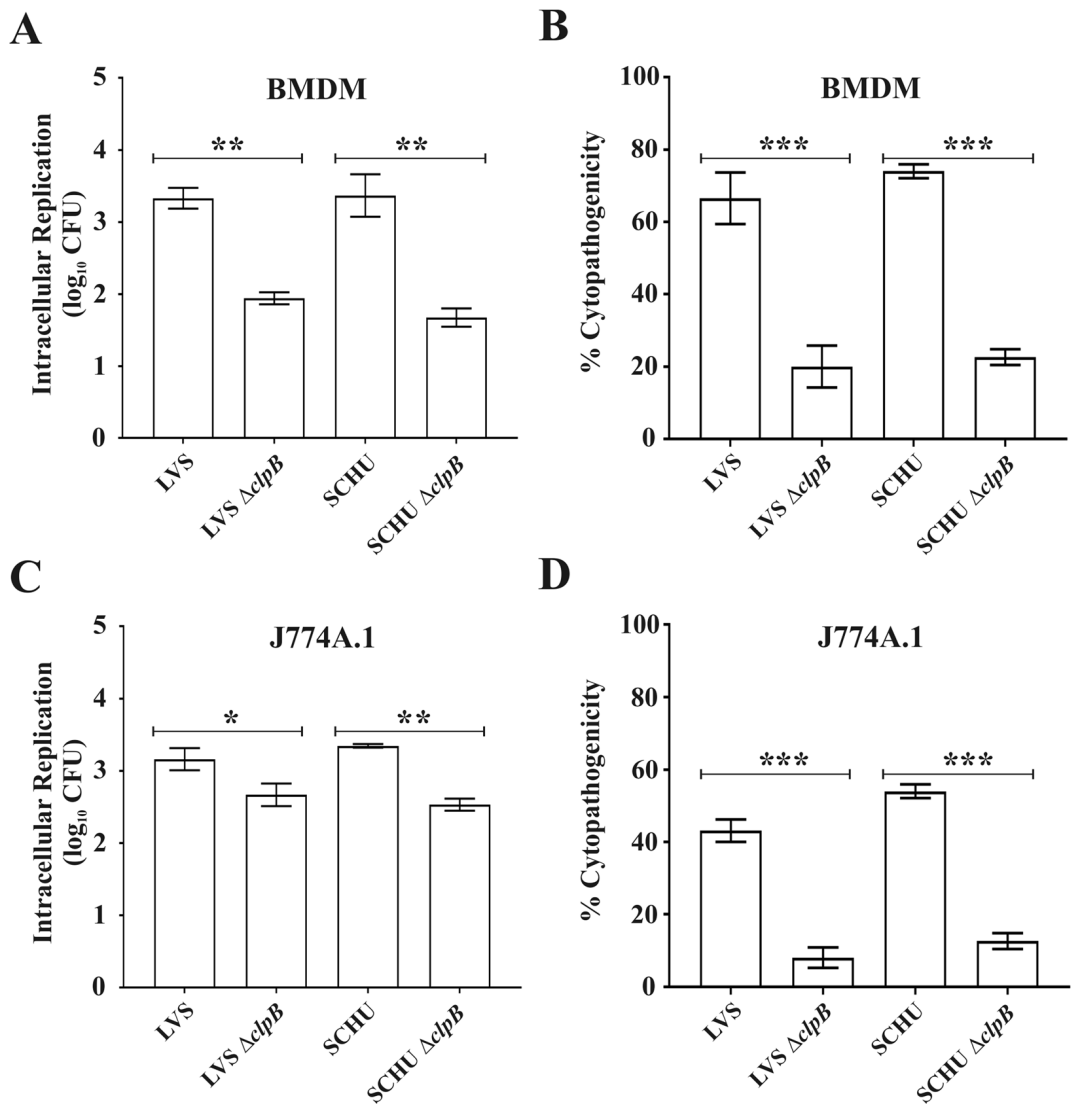
Intracellular growth and LDH release from *F*. *tularensis*-infected BMDM and J774A.1 cells. Growth of the LVS or SCHU S4 Δ*clpB* mutants and the corresponding wild type strains were analyzed by lysis of infected BMDM (**A**) and J774A.1 (**C**) cells at 0 h and 18 h and the number of CFU determined. The net growth mean values ± SEM of at least three independent experiments are shown. Supernatants of cultures of BMDM (**B**) and J774A.1 (**D**) cells infected with the indicated strains were harvested at 18 h. The activity was expressed as a percentage of the level of uninfected lysed cells (positive lysis control). The mean values ± SEM of triplicate wells from one representative experiment of two are shown. The asterisk indicates that the values of the *∆clpB* mutants differed significantly compared to wild type strains (*P < 0.05; **P < 0.01; ***P < 0.001). Unpaired t-test with Welch’s correction was used to compare values.

To determine whether the *clpB* mutation conferred compromised ability to replicate intracellularly, we investigated the ability of the LVS, or SCHU S4 Δ*clpB* mutants to multiply within murine BMDM or the macrophage-like cell line J774 for 24 h. All mutants showed significant replication in BMDM, approximately 100-fold, however, compared to each of the wild-type strains, the replication was significantly compromised (*P* < 0.01 *vs*. the corresponding wild-type strain; Fig. 2A). The absolute differences between mutants and wild-type strains were less marked in J774 cells; however, they were still significant (Fig. 2C). The cytopathogenic effects were very similar between the Δ*clpB* mutants, but much lower (*P* < 0.001) for the mutants than the effects seen upon infection with LVS or SCHU S4 (Fig. 2B,D). This suggests that the deletion of *clpB* is important, although not essential, for the intracellular replication and cytopathogenic effects.

### The LVS and SCHU S4 Δ*clpB* mutants show intact phagosomal escape

**Figure 3:**
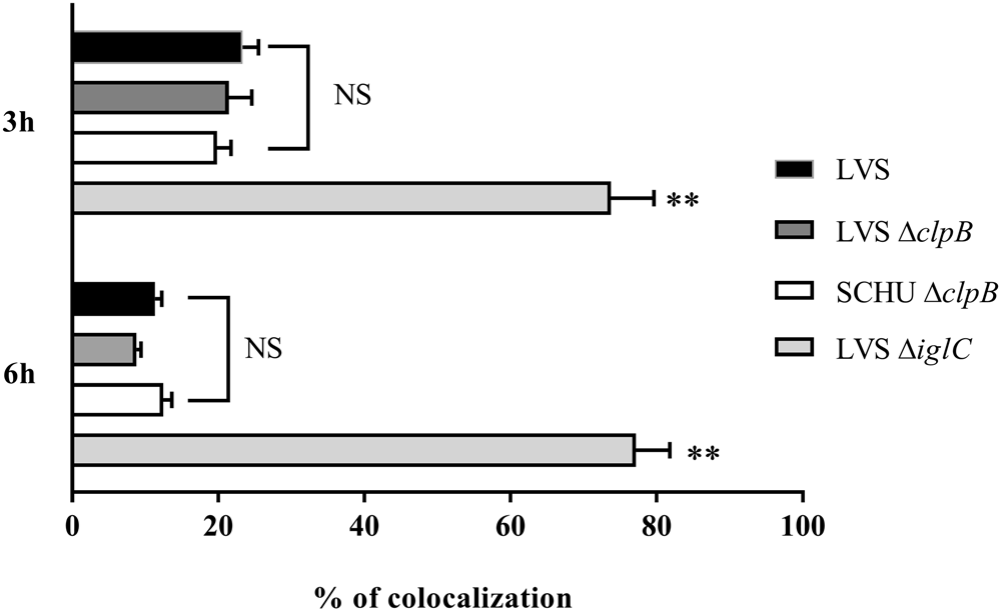
Co-localization of GFP-expressing strains of *F*. *tularensis* with LAMP-1. BMDM cells were infected with the indicated strains expressing GFP for 2 h at an MOI of 30. After 3 and 6 h, specimens were labeled for the late endosomal and lysosomal marker LAMP-1. At least 100 infected cells from multiple cover slips were examined in each experiment. Co-localization was analyzed using a fluorescence microscope (Nikon Eclipse 90i, Nikon, Japan). Results are representative of two experiments and presented as % of co-localization of GFP-expressing bacteria with LAMP-1. The asterisks indicate that the values of the LVS *∆iglC* mutant differed significantly compared to LVS at 3 h and 6 h (**P < 0.01); however, the differences between LVS and LVS *∆clpB* or SCHU *∆clpB* were non-significant (NS *P* > 0.05*)*. Unpaired t-test with Welch’s correction was used to compare values.

In view of the compromised ability of the Δ*clpB* mutants to replicate intracellularly, we investigated whether the mutants also showed compromised phagosomal escape, since the two phenotypes are intimately linked for FPI mutants, *i*.*e*., those that are defective for the escape from the phagosomal compartment also lack intracellular replication^3^. The most common marker used to investigate the intraphagosomal localization by immunofluorescence is LAMP-1, which is a late endosomal and lysosomal marker acquired within 30 min by the *Francisella*-containing phagosome^48,49^. Thus, we compared the degree of co-localization in BMDM between LAMP and the SCHU S4 and LVS strains, or the corresponding Δ*clpB* mutants, each expressing the green fluorescent protein (GFP), at 3 h and 6 h. As control, the Δ*iglC* mutant of LVS, which lacks phagosomal escape, was used. At both the 3 and 6 h time points, there were no significant differences between the LVS and the Δ*clpB* mutant strains, demonstrating a normal phagosomal escape of the two mutants (Fig. 3).

**Figure 4:**
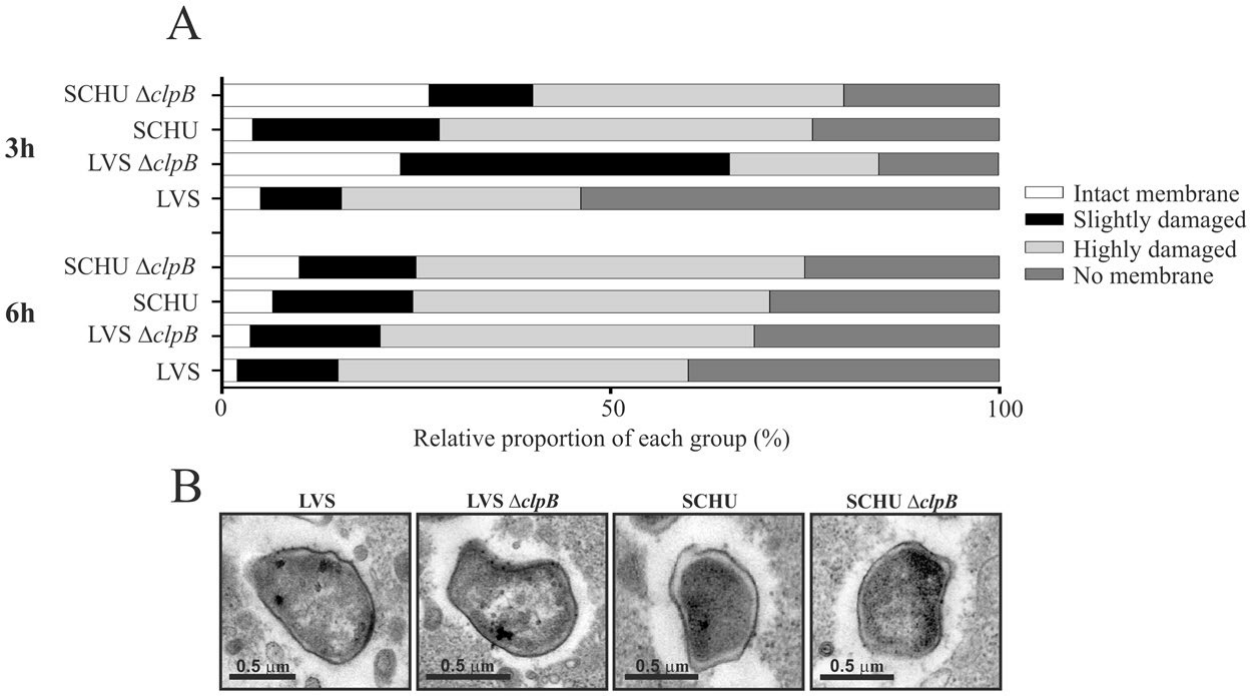
Assessment of the phagosomal membrane integrity of BMDM cells infected with indicated *F*. *tularensis* strains by the TEM assay. BMDM cells were infected for 2 h at an MOI of 1,000, washed, further incubated for 3 and 6 h, and then processed for TEM. To examine the membrane integrity of phagosome, at least 100 bacteria from different sections were analyzed for each time point and categorized as follows: Intact, >90% of the phagosomal membrane intact; Slightly damaged, 50–90% of the membrane intact; Highly damaged, 10–50% of the membrane intact; No membrane, <10% of the membrane intact.

To further corroborate the results regarding the phagosomal escape, we also performed transmission electron microscopy. BMDM were infected with SCHU S4, LVS, or the respective Δ*clp*B mutants. The phagosomal membranes were more intact in cells infected with the Δ*clpB* mutants at the 3 h time point, but there were no significant differences between the SCHU S4 and LVS strains and the Δ*clpB* mutants at the 6 h time point (Fig. 4A). Representative images are shown in Fig. 4B).

Collectively, the findings using the two assays to determine phagosomal escape demonstrate similar results and the SCHU S4 and LVS Δ*clpB* mutant exhibited essentially intact phagosomal escape.

### The *F. tularensis* Δ*clpB* mutants show compromised T6S

We and others have previously reported that the Δ*clpB* null mutants of *F*. *tularensis* SCHU S4 LVS, and a clinical isolate of *holarctica* are highly attenuated *in vivo*^39,40,42,50^ and we hypothesized that a possible mechanism may be impairment of the T6S. Therefore, the secretion of FPI proteins was investigated using the TEM-1 β-lactamase secretion assay, which is a sensitive method to assess intracellular protein secretion in general and FPI secretion specifically^25,51^. Therefore, TEM-1-fusions of IglC, IglE and VgrG, all FPI proteins known to be secreted, were expressed in LVS, SCHU *∆clp*B and LVS *∆clpB* and, in addition, IglE-FS1, a frame-shift mutant of IglE (first 2 to 6 amino acids), since it is secreted at much higher levels compared to the wild-type IglE^52^. The SCHU S4 strain could not be used because of biosafety restrictions; instead, a mutant, Δ*ggt* was used. SCHU Δ*ggt* is highly attenuated and deficient in the gamma-glutamyltranspeptidase (*ggt*), essential for the utilization of glutathione; however, the presence of cysteine allows it to replicate intracellularly^53^.

**Figure 5:**
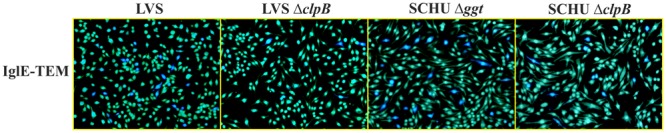
Representative examples of secretion of β-lactamase-tagged IglE protein in J774A.1 macrophages. Macrophages were infected either with LVS, LVS *∆clp*B, SCHU *∆ggt* or SCHU *∆clp*B expressing IglE-TEM fusions for 18 h and the fluorescence was determined. TEM β-lactamase activity was identified by the blue fluorescence emitted by the cleaved CCF2 product, whereas uncleaved CCF2 substrate emitted a green fluorescence. The experiments were repeated at least 4 times using duplicate samples. Representative images of each of 4 samples are shown.

J774 macrophages cells were infected with the aforementioned strains expressing the β-lactamase fusions and translocation of the β-lactamase chimeras was assessed at 18 h after start of infection. The proportion of cells infected with LVS expressing fusions to IglC, IglE, VgrG, or IglE-FS1 demonstrating blue fluorescence ranged from 3.0 to 38.3% for LVS, depending on the fusion analyzed, similar to previously reported values^54^, and from 12.3 to 48.0% for SCHU S4, whereas both *∆clpB* mutants demonstrated very low levels, from 0.3 to 2.9% for the LVS mutant and from 2.5 to 9.0% for the SCHU S4 mutant (Table 1). The lower values for each *∆clpB* mutant were highly significant for each of the constructs (*P* < 0.001), demonstrating that ClpB of both subspecies plays a very important role for efficient T6S during infection. Representative images of the IglE fusion are shown (Fig. 5)

**Table 1:** Secretion of FPI-TEM fusions upon infection of *F*. *tularensis* to J774A.1 cells.

Strain	Secretion of FPI-β-lactamase fusions			
	LVS	LVS ∆*clpB*	SCHU ∆*ggt*	SCHU ∆*clpB*
IglE-TEM	10.6 ± 1.0^a^	0.4 ± 0.2***	14.9 ± 1.9	5.4 ± 0.8***
FS1-TEM	38.3 ± 2.8	2.9 ± 0.3***	48.0 ± 3.9	9.0 ± 0.1***
IglC-TEM	8.7 ± 1.1	0.3 ± 0.1***	14.0 ± 2.5	2.5 ± 0.2***
VgrG-TEM	3.0 ± 0.5	0.7 ± 0.2***	12.3 ± 1.3	4.3 ± 0.4***

^a^The percentages of blue fluorescent cells (indicating secretion of the β-lactamase fused protein) after 18 h of infection was determined by live-cell microscopy.

*** indicates that *P* < 0.001 when each value was compared to the corresponding value for the parental strain.

To confirm that the effect of the *clpB* mutation was specifically related to T6S and not to a general defect in FPI protein expression, Western blot analysis was performed using antibodies against IglA, IglB, IglC, IglD, IglE, IglH, PdpA, PdpB, PdpC, and VgrG. Lysates of LVS and SCHU and their corresponding *∆clpB* mutants were analyzed and no differences were observed between the wild-type strain and the respective *∆clpB* mutant (Fig. S1), demonstrating that the T6S defect observed in the mutants was due to a specific secretion defect and not due to impaired FPI expression.

### ClpB markedly affects the virulence of *F*. *tularensis*

The attenuated phenotypes observed for the Δ*clpB* mutants with respect to phagosomal escape, LDH release, intracellular replication and T6S suggested that they would also show attenuated phenotypes *in vivo*. This has been confirmed previously with regard to the Δ*clpB* mutant of SCHU S4, which has been studied in much detail^39,42^. The SCHU S4 strain is highly virulent in mice with a LD_50_ below 10 CFU^55^.

**Figure 6:**
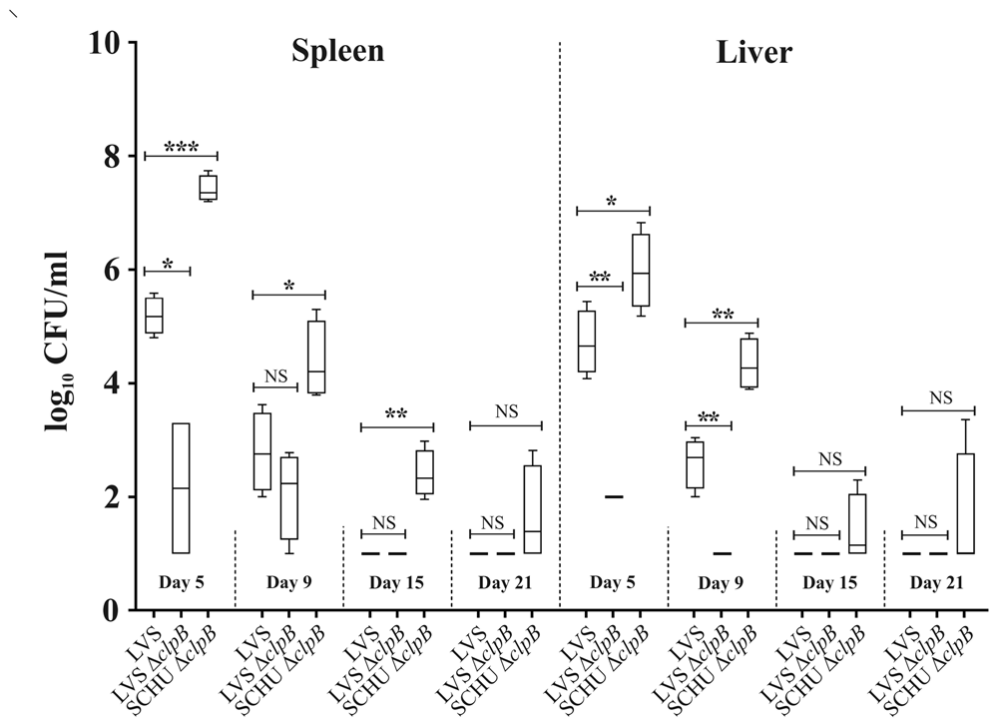
Replication of *F*. *tularensis* strains in liver and spleen following infection of mice. After intradermal inoculation with 1 × 10^4^ CFU of the indicated strain, mice were sacrificed on day 5, 9, 15 and 21, and bacterial burdens in spleen and liver were determined. The mean ± SEM for six mice per group and time point are shown. A significant difference in the bacterial numbers of mutant strains *vs*. LVS is indicated as follows: *P < 0.05; **P < 0.01; ***P < 0.001; NS *P* > 0.05.

To perform a comparative analysis of the *in vivo* phenotype of the Δ*clpB* mutants, mice were subcutaneously infected with the LVS strain or the Δ*clpB* mutants of SCHU S4 or LVS followed by enumeration of bacteria in spleen and liver, the main target organs of tularemia, after an inoculum of approximately 1 × 10^4^ CFU. The bacterial numbers were highest on day 5 and thereafter diminished and were barely detectable on days 15 and 21 (Fig. 6). Regardless of time point, numbers of the LVS Δ*clpB* mutant were low or non-detectable. In contrast, the SCHU S4 Δ*clpB* mutant reached high numbers, significantly higher than the LVS strain, on days 5 and 9. For example, on day 5, the bacterial numbers were 2 log_10_ higher in spleen of the former strain. In separate experiments, the LD_50_ values were estimated and, despite the much higher bacterial numbers of the SCHU S4 Δ*clpB* mutant, its LD_50_ value was in fact higher than that of LVS, approximately 1 × 10^7^ *vs*. 1 × 10^6^ CFU (Fig. S2). These values are in agreement with previously reported ones^42,56^.

Taken together, these results clearly demonstrate that ClpB plays a critical role for the virulence in subspecies *holarctica* and, notably, that the SCHU S4 Δ*clpB* mutant demonstrates more rapid replication that does the LVS strain, despite that the LD_50_ value of the former is higher.

## Discussion

The virulence of *F*. *tularensis* is intimately linked to its intracellular replication and a prerequisite for this is an intact T6SS, encoded by the *Francisella* pathogenicity island^3,19^. There has been much progress regarding the structure of this atypical T6SS. Recent studies have revealed that *F*. *novicida* T6SS assembles on the bacterial cell poles *in vitro* as well as during macrophage infection and constitutes a sheath nearly as long as the bacterial length with a mesh-like architecture, consisting of IglA/IglB^14,23^. ClpB was found to be specifically co-localized with the contracted sheath and was required for the disassembly, in the same way as described for ClpV in other T6SS-containing bacteria^18,23,37^. It should be noted, that there are other bacteria besides *Francisella* that lack the ClpV homologue, but still have functional T6S, *e*.*g*., *Campylobacter*, and *Salmonella*^30–33^. In view of this information and the present findings on *Francisella*, it is likely that ClpB, besides being critical for heat shock survival, also serves a critical role for T6S of those bacteria lacking ClpV. T6SS of several enterobacteria rely on the ATPases ClpV and IcmF for the energy required for the function of the T6SS^28,29^, but since *F*. *tularensis* lacks both ClpV and the Walker A motif in IcmF, which is critical for ATPase activity^22^, it is, therefore, possible that in the absence of ClpV, ClpB, besides contributing to sheath disassembly, generates the energy required for T6S. However, in view of the fact that a low level of secretion was observed in each Δ*clpB* mutant, the contribution, if any, of ClpB to generate energy is dispensable.

In the present study, we demonstrate that, as expected, and in agreement with previous data^23,50^, ClpB displays an essential role for the heat shock survival of subspecies *holarctica* and *tularensis*. There is precedence for the involvement of the ClpB-DnaK disaggregation complex in the assembly of the T6SS, since the insertion of a transmembrane segment within the essential T6SS component TssL of *E*. *coli*, is modulated by DnaK^57^. The findings regarding the LVS Δ*clpB* mutant concur with previously published data^50^.

To further characterize the intracellular phenotypes of the Δ*clpB* mutants, we also investigated their phagosomal escape. Normally, there is close correlation between the intracellular replication and phagosomal escape, as evidenced by numerous findings on various FPI mutants that all lack phagosomal escape and do not replicate intracellularly^3^. We observed that the Δ*clpB* mutants of SCHU S4 and LVS escaped as quickly as did the wild-type strains and, therefore, this was not a cause for their impaired replication. The escape of the LVS mutant has been studied previously and although the degree of escape was not quantified, it was concluded that it displayed a cytoplasmic localization^50^. We have previously demonstrated that FPI mutants lacking T6S, *e*.*g*., Δ*iglA* and Δ*iglC*, replicated at least as rapidly as LVS upon microinjection, regardless of cell type^58^. Therefore, it was concluded that the critical function of the T6SS is to execute phagosomal escape, but it is not essential for the cytoplasmic replication. Based on this hypothesis, the much compromised T6S of the Δ*clpB* mutants may not result in defective intracellular replication and an explanation for the compromised intracellular replication may be the exquisite susceptibility to low pH of both Δ*clpB* mutants, also previously demonstrated for the LVS mutant^50^. In further support of the hypothesis, it was observed in the latter study and also in the present study that the importance of ClpB varied depending on the host cell type. It is likely that the stress encountered, and thereby the relative contribution of ClpB, is not as severe in certain cell lines, such as the J774 macrophage-like cells, as in *ex vivo*-derived cells, such as BMDM.

The two Δ*clpB* mutants showed very low secretion of IglC and other FPI proteins. Previous studies have consistently demonstrated the need for a functional T6S for phagosomal escape, however, the minimal T6S of the Δ*clpB* mutants of *F*. *tularensis* SCHU S4 and LVS demonstrated that even very low levels of T6S are sufficient for the bacteria to escape. In fact, the two mutants showed rather distinct T6S, since the levels observed using the β-lactamase secretion assay were very low for the LVS mutant, whereas the SCHU S4 mutant demonstrated levels distinctly lower than the wild-type strains, but still much higher than the LVS mutant. Despite these differences, there were no obvious differences in their phagosomal escape.

Although both Δ*clpB* mutants demonstrated similar, defective intracellular replication, they showed very distinct phenotypes *in vivo*, since the LVS Δ*clpB* mutant was very compromised and disseminated only marginally to liver and spleen. In contrast, the SCHU S4 Δ*clpB* mutant disseminated and replicated more effectively than did the LVS strain. Paradoxically, the LD_50_ value of the Δ*clpB* mutant was still 10-fold higher. One possible explanation could be that the lethality of infection is partly dependent on T6S effectors and, therefore, despite the more effective replication of the SCHU S4 Δ*clpB* mutant, it is not as lethal as the LVS strain due to its compromised T6S.

Collectively, our data demonstrate a critical role of ClpB in both of the human-pathogenic subspecies *holarctica* and *tularensis* for the normal T6S. The Δ*clpB* mutants also demonstrate defective intracellular replication and virulence and also impaired handling of stress stimuli, such as heat shock and low pH. The exact contribution of ClpB and the T6SS to all of the observed phenotypes is still unclear and it will be important in future studies to elucidate this, since it will provide essential information about the control and regulation of the T6SS of *Francisella* and possibly other T6SS that lack ClpV.

## Methods

### Bacterial strains and growth conditions

The bacterial strains and plasmids used in this study are listed in Table S1. *F*. *tularensis* strains were cultured either in Tryptic soy broth (TSB) supplemented with 0.1% cysteine (w/v) and 0.1% glucose (w/v) or in chamberlain defined medium (CDM). Kanamycin (10 μg/ml) was added to the medium when needed. All bacteriological work related to the SCHU S4 strain was carried out in a biosafety level 3 facility certified by the Swedish Work Environment Authority. As decided by the Swedish Work Environment Authority, The SCHU S4 Δ*clpB* and Δ*ggt* mutants are classified as BSL2 agents.

### Cultivation and infection of macrophages

The J774A.1 mouse macrophage-like cell line (ATCC TIB-67) or bone marrow-derived macrophages (BMDMs) were used in the cell infection assays. J774A.1 macrophages were cultured and maintained in DMEM (GIBCO BRL, Grand Island, NY, USA) with 10% heat-inactivated FBS (GIBCO). BMDMs were isolated by flushing bone marrow cells from the femurs and tibias of C57BL/6 mice as described previously^59^. These cells were cultured for 4 days in DMEM containing 10% FBS, 5 μg/ml gentamicin and 20% conditioned media (CM) from L929 cells (ATCC no CCL-1) overexpressing M-CSF, after which they were grown in medium lacking gentamicin. Macrophages were seeded in tissue culture plates in DMEM with 10% FBS and incubation overnight. A multiplicity of infection (MOI) of 200 was used in all infection experiments, with the exception of the TEM study, where an MOI of 1,000 was used. After 24 h post infection, the macrophage monolayers were lysed in 0.1% deoxycholate, serially diluted plated on agar plates for determination of viable counts.

### Lactate dehydrogenase (LDH) release assay

At indicated time points, culture supernatant were collected from infected wells and the release of LDH, indicative of cytopathogenicity, determined using the LDH assay kit according to the manufacturer’s instructions (Promega, Madison, WI). Uninfected J774A.1 cells lysed in 0.1% deoxycholate served as a positive control, and the value for this control was arbitrarily considered as 100% cell lysis. Samples absorbance was expressed as a percentage of the positive control value. The assay was performed with triplicate samples and was repeated at least three times.

### Temperature and pH susceptibility test

Bacteria were grown overnight on modified GC-agar, resuspended in Chamberlain to give an OD_600_ of 0.5, and 0.25 ml of the culture was placed in a 2 ml Eppendorf tube and placed statically in a water bath at 50 °C for 30 min. The tubes were then placed on ice, serially diluted in PBS and plated on agar plates to determine the number of viable bacteria. To determine the sensitivity to pH stress, overnight agar-grown bacteria were resuspended in TSB, pH 4.5 and incubated at 37 °C for 1 h. At indicated time points, aliquots were sampled, serially diluted in PBS and plated to determine the number of viable bacteria.

### Paraquat and peroxynitrite susceptibility assay

Susceptibility of *F*. *tularensis* strains to O_2_^−^ was determined by use of the O_2_^−^ generating compound paraquat dichloride hydrate (Sigma-Aldrich, St. Louis, USA) in a disc diffusion assay as previously described^47^. Susceptibility to peroxynitrite was tested as previously described after incubation for 3 h in the presence of 0.40 mM SIN-1^47^.

### β-lactamase secretion assay

The TEM1 β-lactamase secretion assay was performed essentially following the same protocol as described before^25^. In brief, 1.5 × 10^5^ J774 cells/well were seeded onto BD Falcon 8-wells glass chambers slides (BD Biosciences, Bedford, MA, USA) and incubated overnight before infected with the indicated bacterial strain. After 2 h of infection, cells were washed and incubated further with 5 µg/ml of gentamicin (defined as time point zero). After 30 min, cells were washed and after an additional 18 h, washed and loaded with CCF2/AM (Invitrogen, CA, USA) and Probenicid (Sigma). Translocation of β-lactamase fusions was determined with a live-cell imaging microscope (Nikon Eclipse Ti-E) equipped with a Nikon DS-U2/L2 camera, using a Chroma beta-lactamase double filter. For statistical analysis of blue *vs*, green fluorescent cells, an average of 3,000–6,000 cells that included pictures from three experiments for a given strain was counted.

### Intracellular immunofluorescence assay

To assess phagosomal escape of GFP-expressing *∆clpB* mutants of *F*. *tularensis*, the *∆clpB* mutants (all expressing pKK289Km-GFP) were used in the cell infections as described previously^21^. As negative controls, LVS *∆iglC*^60^ was used. At indicated time points, cells were fixed, and stained using an antibody recognizing the LAMP-1 glycoprotein (BD Pharmingen, San Jose, CA). Colocalization of GFP-labeled *F*. *tularensis* and LAMP-1 was analyzed with an epifluorescence microscope (Nikon Eclipse 90i, Nikon Instruments Europe BV, Netherlands). Two biological replicates were used and a minimum of 100 bacteria per strain and time point were scored.

### Transmission electron microscopy

The analysis was carried out as previously described^54^. BMDM cells were infected for 2 h, washed, and incubated for 3 h or 6 h. Monolayers were washed and fixed with 2.5% glutaraldehyde, washed in 0.1 M sodium cacodylate buffer, scraped, and treated with 1% osmium tetroxide. Embedded cell pellets were cut into 70 nm sections and stained with uranyl acetate and lead citrate before analyzed with a JEM 1230 transmission electron microscope (Jeol Ltd., Tokyo, Japan). At least 100 bacteria from different sections were analyzed for each time point and categorized as having (i) an intact phagosomal membrane, (ii) a slightly damaged phagosomal membrane (<50% of membrane integrity affected), (iii) a highly damaged phagosomal membrane (>50% of membrane integrity affected), or (iv) no residual membrane.

### Western blot analysis

The samples were dissolved in sample buffer, boiled before analyzed applied on a 12% sodium dodecyl sulfate-polyacrylamide gel electrophoresis (SDS-PAGE) and transferred onto a nitrocellulose membrane. Blots were probed by either rabbit polyclonal antibodies against IglA, PdpC (BEI Resources), IglH and VgrG, (Inbiolabs, Tallinn, Estonia) or mouse monoclonal antibodies against IglB, IglC, IglD, PdpA and pdpB (BEI Resources). To probe against IglE^52^, rat polyclonal antibodies was used. Polyclonal IgY chicken antibodies, specific to FupA was used (Agrisera).

### Mouse infection

For determination of the killing capacity of each strain, C57BL/6 female mice (n = 6) were infected intradermally with approximately 1 × 10^4^ CFU for each *F*. *tularensis* strain. Mice were examined twice daily and euthanized if they displayed signs of severe disease. At days 5, 9, 15 and 21, infected mice were killed and serial dilutions of the homogenized organs were plated. The animal experiments were approved by the local Ethical Committee on Laboratory Animals, Umeå, Sweden (approval no. A67-14) and all experiments were performed in accordance to the national guidelines and regulations.

### Statistical analysis

For the statistical analysis, GraphPad Prism 7 (GraphPad Software Inc., CA, USA) was used.

## Electronic supplementary material

Supplementary table and figures
