# Supplementary material for: ClpB mutants of Francisella tularensis subspecies holarctica and tularensis are defective for type VI secretion and intracellular replication
Source: Sci Rep. 2018 Jul 27;8:11324. doi: 10.1038/s41598-018-29745-4 (PMC6063899; doi:10.1038/s41598-018-29745-4)
Supplement: Supplementary file 1 — Supplementary table and figures [file 41598_2018_29745_MOESM1_ESM.pdf]

Supplementary Information

ClpB mutants of *Francisella tularensis* subspecies *holarctica* and *tularensis* are defective for type VI secretion and intracellular replication

Athar Alam, Igor Golovliov, Eram Javed, Anders Sjöstedt*

Department of Clinical Microbiology, Umeå University, SE-901 85 Umeå, Sweden.

***Address correspondence to**: Anders.Sjostedt@umu.se.

This file includes:

1. Supplementary Table.1
2. Supplementary Fig.S1
3. Supplementary Fig.S2

Table S1. Strains and plasmids used in this study

Strain or plasmid	Relevant genotype and/or phenotype	Source or reference
***F. tularensis* strains**		
LVS	Live vaccine strain	FSC[a]
LVS *ΔclpB*	Live vaccine strain, in-frame deletion of *clpB*	1
LVS *ΔiglC*	Live vaccine strain, in-frame deletion of *iglC*	2
SCHU S4	*Francisella tularensis* subsp. *tularensis*	FSC
SCHU S4 *ΔclpB*	*Francisella tularensis* subsp. *tularensis*, in-frame deletion of *clpB*	3
SCHU S4 *Δggt*	*Francisella tularensis* subsp. *tularensis*, in-frame deletion of γ-glutamyltransferase (*ggt)*	4
**Plasmids**		
pKK289-GFP	Expression plasmid carrying a *gfp* gene under the control of LVS GroESL promoter, Tet^r	5
pJEB709	pMOL42 derivative encoding mature TEM β-lactamase from *E. coli*, Km^R	6
pJEB733	pJEB709, encoding IglC-TEM, Km^R	6
pSK003	pJEB709, encoding IglE-TEM, Km^R	6
pJEB953	pJEB709 encoding IglE $_{FS1(2–6)}$-TEM, Km^R	7
pMOL95	pJEB709, encoding VgrG-TEM, Km^R	6

[a] Obtained from the *Francisella* Strain Collection, Swedish Defence Research Agency, Umeå, Sweden,

References

1 Barrigan, L. M. *et al.* Infection with *Francisella tularensis* LVS *clpB* leads to an altered yet protective immune response. *Infect Immun* 81, 2028-2042, doi:10.1128/IAI.00207-13 (2013).

2 Golovliov, I., Sjöstedt, A., Mokrievich, A. & Pavlov, V. A method for allelic replacement in *Francisella tularensis*. *FEMS Microbiol Lett* 222, 273-280. (2003).

3 Conlan, J. W. *et al.* Differential ability of novel attenuated targeted deletion mutants of *Francisella tularensis* subspecies *tularensis* strain SCHU S4 to protect mice against aerosol challenge with virulent bacteria: Effects of host background and route of immunization. *Vaccine* 28, 1824-1831, doi:S0264-410X(09)01904-5 [pii]

4 Ireland, P. M., LeButt, H., Thomas, R. M. & Oyston, P. C. A *Francisella tularensis* SCHU S4 mutant deficient in gamma-glutamyltransferase activity induces protective immunity: characterization of an attenuated vaccine candidate. *Microbiology* 157, 3172-3179, doi:10.1099/mic.0.052902-0

5 Bönquist, L., Lindgren, H., Golovliov, I., Guina, T. & Sjöstedt, A. MglA and Igl proteins contribute to the modulation of *Francisella tularensis* live vaccine strain-containing phagosomes in murine macrophages. *Infect Immun* 76, 3502-3510, doi:10.1128/IAI.00226-08

6 Bröms, J. E., Meyer, L., Sun, K., Lavander, M. & Sjöstedt, A. Unique substrates secreted by the type VI secretion system of *Francisella tularensis* during intramacrophage infection. *PLoS One* 7, e50473, doi:10.1371/journal.pone.0050473

7 Bröms, J. E., Meyer, L. & Sjöstedt, A. A mutagenesis-based approach identifies amino acids in the N-terminal part of *Francisella tularensis* IglE that critically control Type VI system-mediated secretion. *Virulence*, 1-27, doi:10.1080/21505594.2016.1258507 (2016).

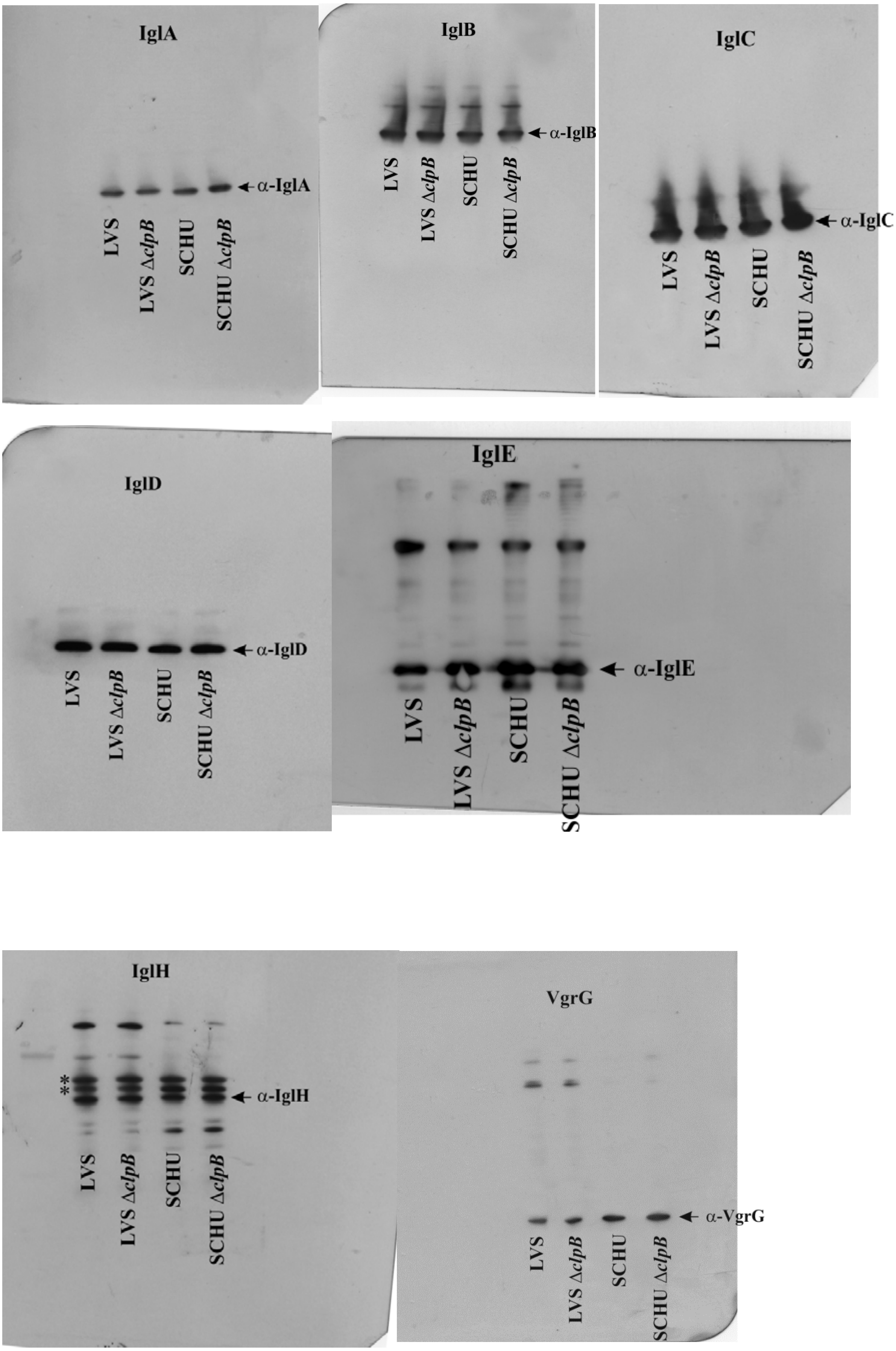
IglA
α-IglA
LVS
LVS ΔclpB
SCHU
SCHU ΔclpB
IglB
α-IglB
LVS
LVS ΔclpB
SCHU
SCHU ΔclpB
IglC
α-IglC
LVS
LVS ΔclpB
SCHU
SCHU ΔclpB
IglD
α-IglD
LVS
LVS ΔclpB
SCHU
SCHU ΔclpB
IglE
α-IglE
LVS
LVS ΔclpB
SCHU
SCHU ΔclpB
IglH
**
α-IglH
LVS
LVS ΔclpB
SCHU
SCHU ΔclpB
VgrG
α-VgrG
LVS
LVS ΔclpB
SCHU
SCHU ΔclpB

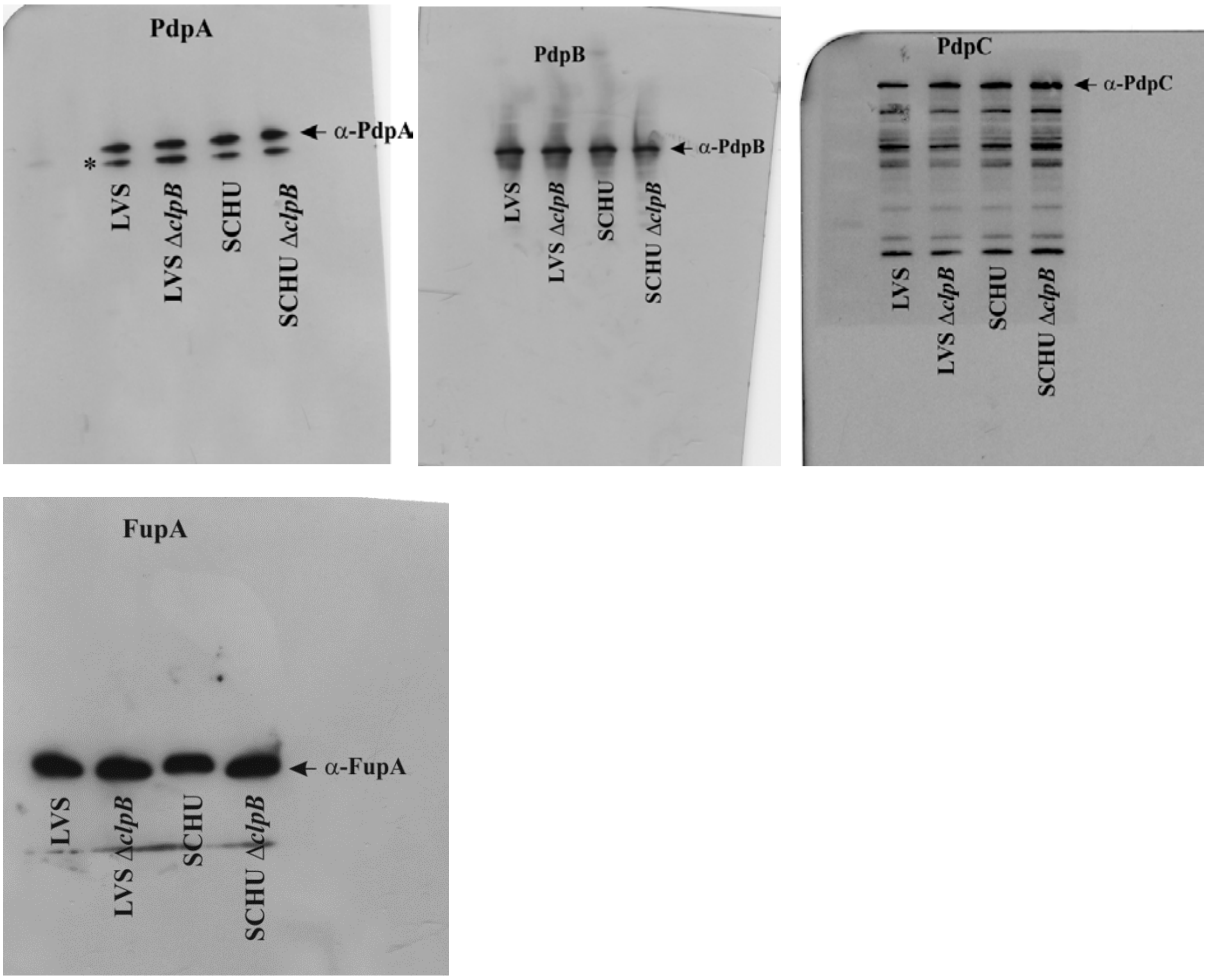

Fig. S1: Western blot analysis of total levels of FPI proteins of the indicated *F. tularensis* strains. Whole cell lysates of each strain were prepared, separated on 12 % SDS-PAGE and probed with specific antibodies against indicated FPI proteins. FupA was used as loading control. Assays were repeated at least twice and representative blots are shown.

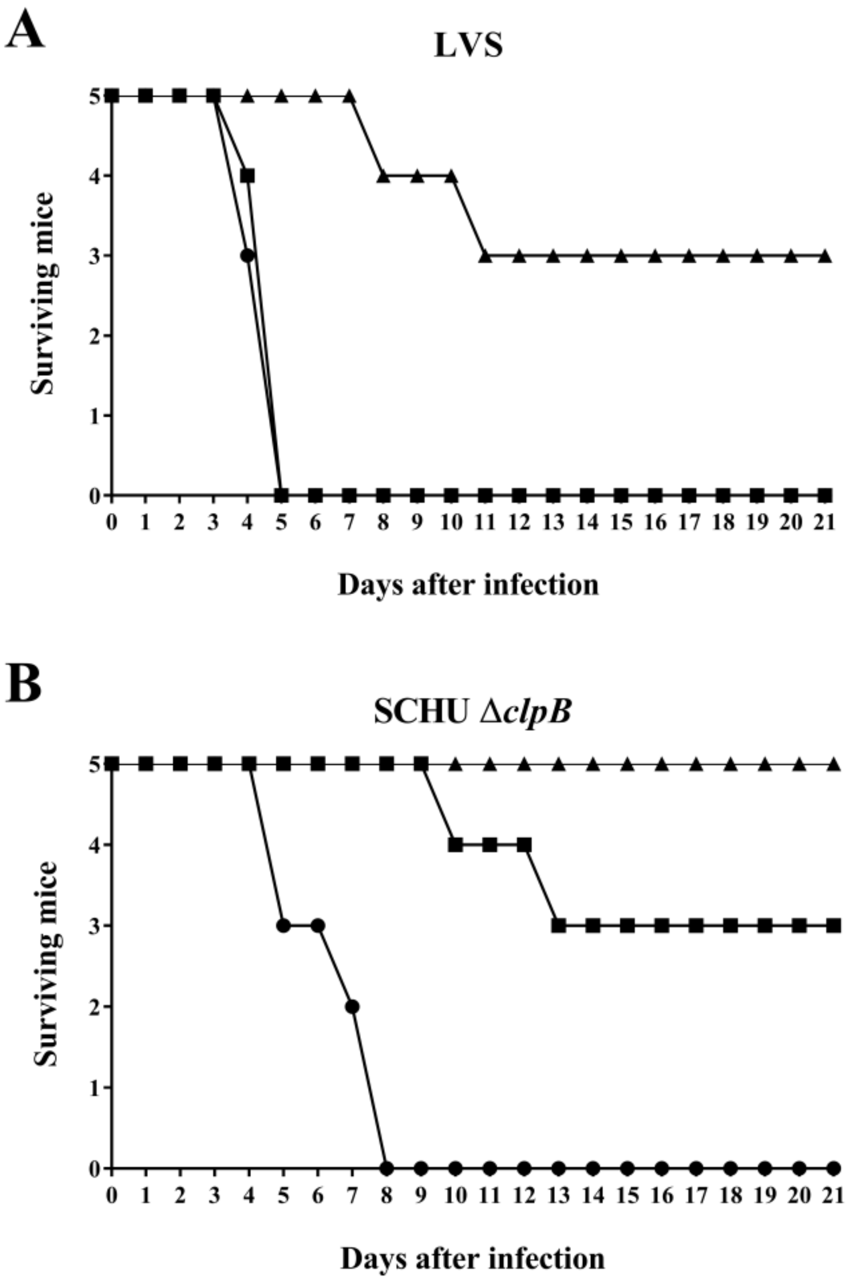

Fig S2. ClpB is required for lethality in C57BL/6 mice. Mice were infected intradermally with 6 x 10^7 CFU (circle), 6 x 10^6 CFU (square), or 7 x 10^5 CFU (triangle) of LVS (A); or with 1 x 10^8 CFU (circle), 8 x 10^6 CFU (square), or 8 x 10^5 CFU (triangle) of SCHU S4 Δ*clpB* (B). Mice were monitored for signs of morbidity for up to 21 d post infection. The data represents one representative experiment out of 3 where groups of 5 (n = 5) mice were used.
